# Beyond the ‘Pregnancy Black Box’: a global roadmap for artificial intelligence-driven pharmacogenomics in maternal-neonatal health

**DOI:** 10.1038/s41397-026-00422-4

**Published:** 2026-06-22

**Authors:** Mohamed A. Ismail

**Affiliations:** Hamad Medical Corporation, Doha, Qatar

**Keywords:** Preclinical research, Translational research

## Abstract

Maternal and neonatal health (MNH) urgently requires precision medicine interventions, as morbidity, mortality, and health disparities hinder the achievement of Sustainable Development Goal 3. Clinical implementation of artificial intelligence (AI)-powered Pharmacogenomics (PGx) requires validated, transparent algorithms and frameworks. The “pregnancy black box”-which refers to a data void due to historical exclusion of pregnant and postpartum women from clinical trials-continues to create bias in AI models. The review establishes a path for upcoming research, including methods to reduce algorithmic bias via AI-driven data augmentation, resolution of ethical challenges, and creation of international registries. Ultimately, leveraging AI for remote monitoring is crucial for enhancing equitable access in lower-resource environments. The proposed roadmap provides organizations with a robust framework to develop AI-driven PGx systems, which will enable safer and more tailored pharmacotherapy for mothers and their newborns.

## Introduction

Maternal deaths and neonatal complications remain high, driven in part by persistent disparities [1], which calls for a shift toward precision- medicine during pregnancy. These physiological shifts are associated with an increased risk of adverse drug reactions (ADRs), underscoring the need for personalized intervention [2]. However, current PGx remains limited by a static framework that does not fully account for such dynamics [3, 4]. Genetic polymorphisms in drug-metabolizing enzymes and transporters drive significant variability in perinatal drug efficacy and safety. Recent evidence from a meta-analysis indicates that while SSRIs show no significant risk after adjusting for depression, SNRIs maintained a significant association with hypertension and preeclampsia [5]. Additionally, candidate gene studies have suggested that variations in OPRM1 and COMT may influence NAS severity, though these findings have not been consistently replicated in larger GWAS [6]. In non-pregnant cohorts, *SLC22A1* polymorphisms diminish metformin’s cellular uptake and glycemic response, suggesting implications for gestational diabetes management [7]. Mapping fetal CYP450 expression provides a foundation for enhancing pregnancy-specific medication safety [8]. Furthermore, machine learning (ML) models that integrate genetic, and environmental datasets improve risk prediction and optimize personalized dosing [8]. However, limited external validation across diverse populations remains a barrier to widespread clinical adoption [9]. A globally coordinated, ethically governed roadmap for AI-driven multi-omics integration is important to dismantle the ‘pregnancy black box’ and achieve equitable precision medicine for MNH within the next decade.

## Methodology: literature search strategy

This state-of-the-art conceptual review [10] was conducted to identify, synthesize, and critically appraise literature concerning the convergence of AI and PGx in MNH. The search targeted literature from 2016 to early 2026, prioritizing results from 2022–2026. The detailed methodology. The detailed methodology, including a comprehensive literature search, specific databases used, and full inclusion and exclusion criteria for this review, is provided in Supplementary File 1.

## The synergy of AI and PGx in maternal-neonatal precision medicine

**Fig. 1:**
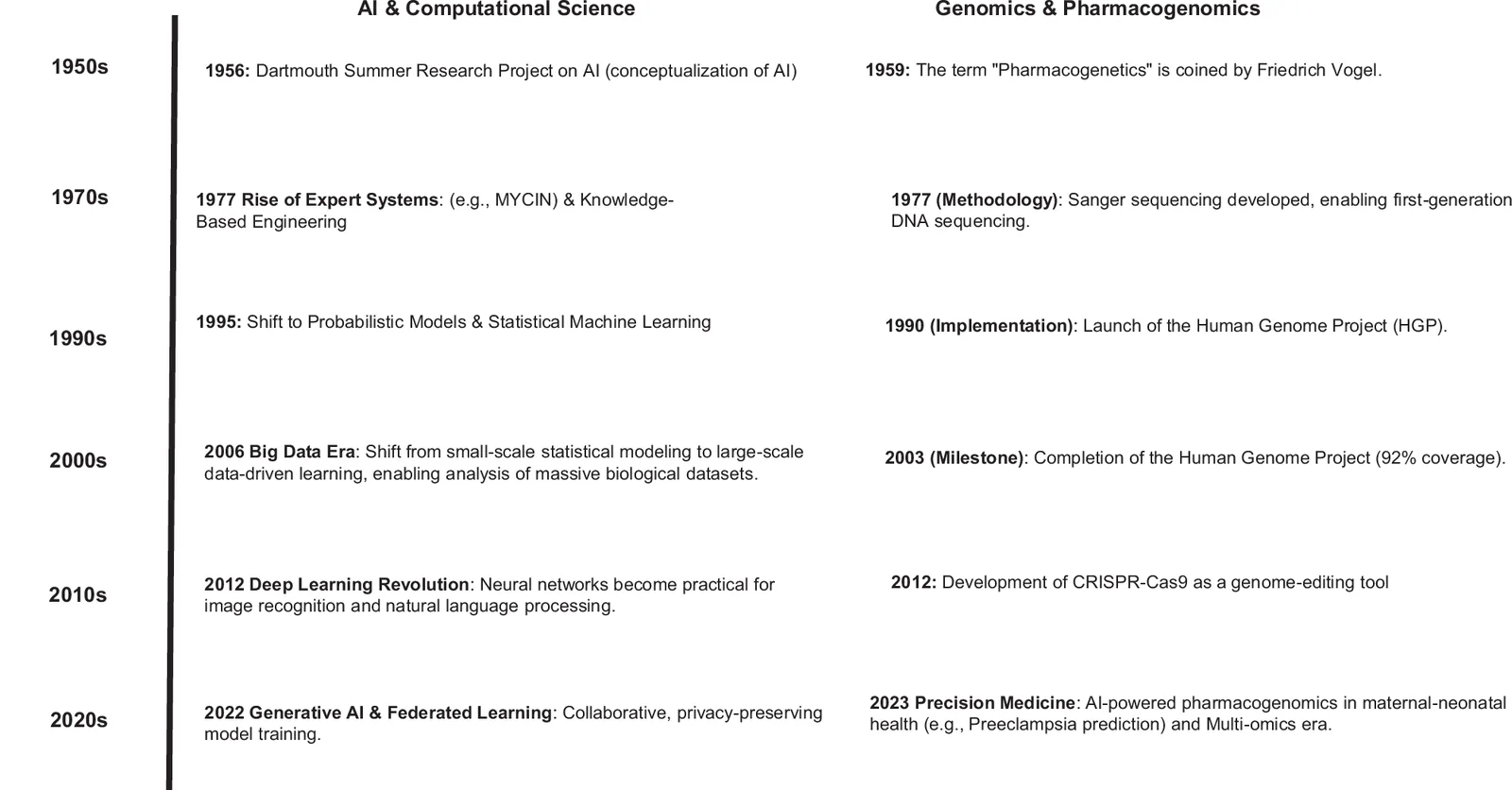
Historical Evolution of Artificial Intelligence and Pharmacogenomics. A chronological timeline starting from early AI concepts and PGx foundations (moving from symbolic logic to probabilistic models), through significant discoveries, technological advancements (e.g., Human Genome Project, rise of ML), and culminating in current AI-powered PGx applications in maternal neonatal care (e.g., preeclampsia prediction, personalized dosing, multi-omics integration, FL). s* Refers to the decade spanning 10 years.

AI’s algorithmic synthesis of high-dimensional genomic and clinical data transforms variable pharmacokinetic profiles into actionable therapeutic insights. The historical development and key milestones in both AI and PGx are shown in (Fig. 1), which highlights their subsequent convergence.

### AI Enhances PGx-Driven risk prediction

Synthesizing disparate multi-omics data and real-time phenotypes allows AI to map the non-linear biological variables unique to pregnancy. ML methods such as support vector machines and random forests predict antidepressant efficacy and adverse reactions by integrating *CYP2D6* status with gestational pharmacokinetic changes [11, 12]. Moreover, ML integration of maternal *OPRM1* polymorphisms and epigenetic markers outperforms traditional clinical evaluations in assessing NAS severity [13].

### Beyond single-omics: AI as a multi-omics integrator

**Fig. 2:**
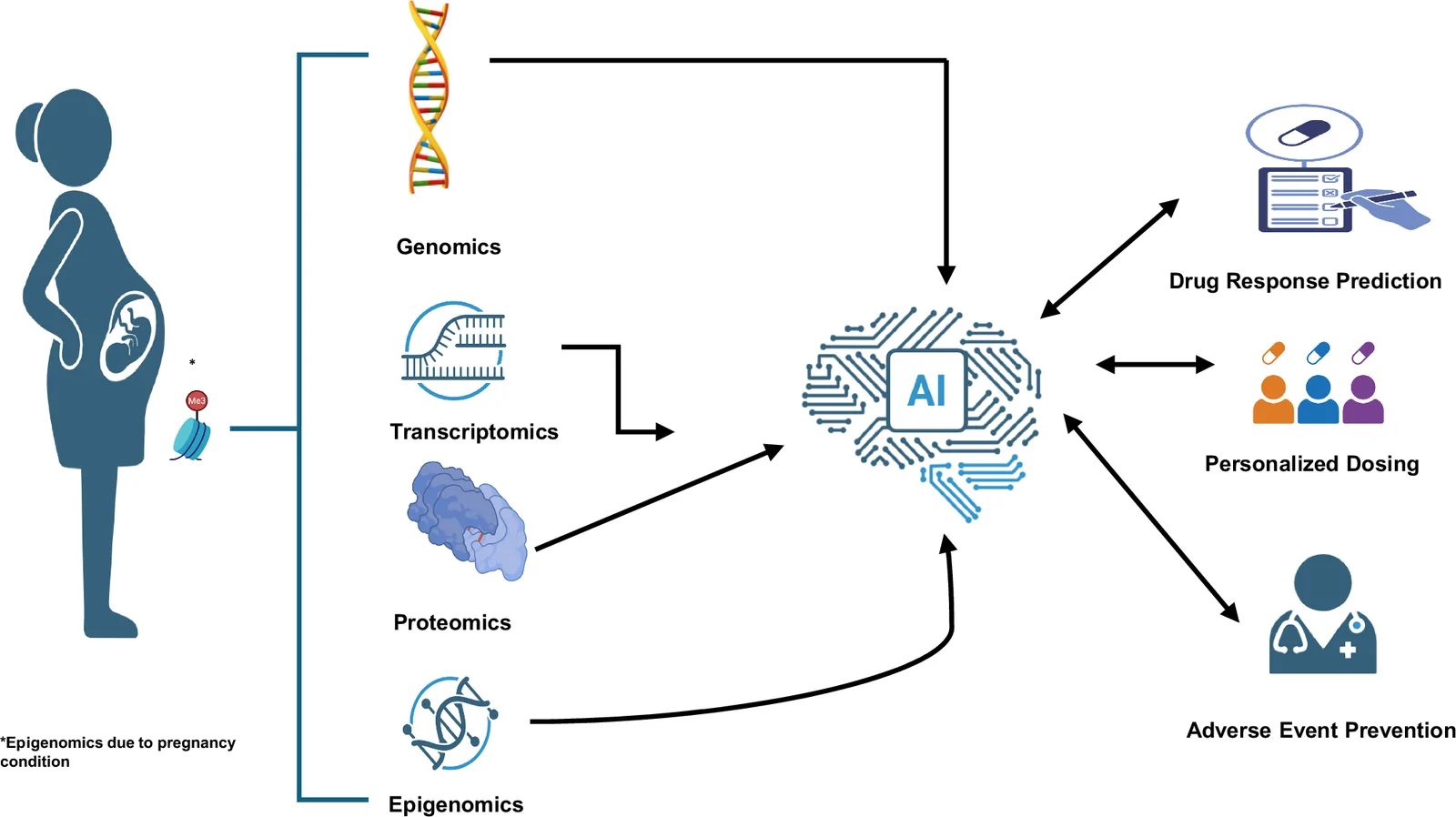
Conceptual framework of AI-powered precision medicine Maternal-Neonatal Health. This schematic demonstrates the integration of diverse biological data including genomics, transcriptomics, proteomics, and epigenomics into AI models to generate actionable clinical outputs such as drug response prediction, personalized dosing, and adverse event prevention.

The dynamic physiological changes of pregnancy underscore the need for multi-omics data integration in perinatal medicine [14]. AI systems employing neural networks can integrate *CYP3A4* variants with protein-level biomarkers, such as the *sFlt-1/PlGF* ratio for holistic risk assessment. By synthesizing these multimodal data layers (Fig. 2), AI models achieve high predictive accuracy, tailoring therapeutic decisions to the unique context of pregnancy [15].

## Current applications of AI in maternal/perinatal PGx

### Drug response prediction

Deep learning (DL) models integrating PGx indicators with trimester-specific metabolic shifts markedly improve SSRI treatment responses [16]. These models predict optimal dose adjustments to potentially decrease the incidence of neonatal withdrawal syndrome. Thus, AI refines drug response prediction by discerning complex interactions between genetic, environmental, and clinical variables.

### Pregnancy complication risk stratification

AI systems analyzing over 200 clinical, metabolomic, and laboratory variables achieve 87.5% sensitivity and 94.1% specificity for early-onset preeclampsia prediction using maternal characteristics, metabolites, and routine lab tests from 11–15 weeks and 6 days of gestation [17]. Likewise, the simulation of fetal drug exposures is advancing through ‘digital twin’ technology, which integrates in vitro microphysiological systems with physics-informed neural networks to model pharmacokinetics with high fidelity [18]. The shift from theoretical modeling to large-scale clinical validation is further demonstrated by recent high-impact initiatives (Table 1), which utilize multi-omics integration to bridge the ‘pregnancy black box’. These models emphasize the transition from reactive observation to proactive, site-specific risk management.

**Table 1:** High-Dimensional AI and Multi-Omic Frameworks for Global Maternal-Neonatal Risk Stratification.

Method Name	Key Layers & Data	AI Method	Use Case	Outcome
MOMI Consortium Pipeline [19].	Genomics, Proteomics, Metabolomics.	Integrative multi-omics pipeline.	Mechanisms for Preterm Birth & Preeclampsia.	Identification of site-specific molecular signatures across global cohorts encompassing over 24,000 pregnancies.
Perinatology Precision AI [20].	Clinical Data + multi-omics.	Machine learning (ANN, RF, SVM).	Identifying risk before clinical onset (e.g., Growth Failure).	Shift from reactive to proactive care by predicting growth failure before it shows on curves.
Neonatal Sepsis ML with Genetic Variants [21]+.	Biochemical markers (PCT, IL‑6) + Genomics (CRP, IL‑10 variants).	SVM, Random Forest.	Neonatal sepsis risk prediction.	Achieved high-accuracy (95.45%) sepsis risk classification and identified PCT, WBC, and IL-6 as key predictive biomarkers.
Multi-scale Genotype-Pheno [22].	Single cell multi-omics.	Transformer‑based generative foundation model (scGPT)	Predicting chemical & genetic perturbations.	Predicts transcriptional responses to chemical and genetic perturbations with high accuracy and generalizes unseen perturbations using self-supervised learning on large-scale single-cell data.
Transformer-based Multi-Omics PTB Predictor [23].	cfDNA + cfRNA (cell-free).	Transformer-based LLM (GeneLLM architecture).	Preterm birth risk prediction from plasma.	Demonstrated superior predictive accuracy (AUC 0.890) compared to single-modality models (*p* < 0.05).
Epigenetic-ML HDP Predictor [24].	First-trimester DNA methylation (CTSA, HMGB1, miR1908/FADS2) + clinical factors.	LASSO + XGBoost + Random Forest ensemble	First-trimester HDP risk prediction.	Validated differentially methylated regions (DMRs) with high predictive performance (AUC 0.863 training, 0.757 validation).
GNN for Placental Insufficiency* [25].	Genomics, Transcriptomics, Proteomics, Metabolomics.	Heterogeneous Graph Neural Network (HGNN) with attention.	Classifying placental insufficiency & folate deficiency.	Achieved 94.7% classification accuracy (AUROC 0.978) and identified significant MTHFR and FOLR molecular signatures.
AI‑Based Preterm Birth Prediction Review** [26].	Multi-omics integration, EHR data, ultrasound images, cervical elastography.	Deep learning, transformer architecture, ML.	Preterm birth risk prediction.	AUC range 0.61–0.89 across modalities; 79% of studies at high risk of bias per PROBAST; median TRIPOD adherence 49%.

This table details large-scale, multi-omic AI initiatives designed to identify systemic risks such as preterm birth, preeclampsia, and neonatal sepsis. + refer to single-center study; external validation lacking. * Model trained on terminal delivery tissues; validated for retrospective diagnostic confirmation and molecular phenotyping, not currently capable of antenatal risk prediction. ** Narrative review of multi-omics studies (not primary research).

### Translating AI-Driven PGx into maternal and neonatal care

Large-scale initiatives increasingly demonstrate the clinical potential of AI-driven PGx in maternal and neonatal care (MNC). UCSF implements comprehensive clinical PGx programs, establishing frameworks for preemptive testing in academic centers [19]. Ongoing clinical trials develop AI-driven, trimester-specific pharmacokinetic frameworks that incorporate *CYP2D6* and *CYP2C19* status to identify metabolic extremes (Table 2), thereby facilitating personalized antidepressant dosing.

**Table 2:** Overview of AI-Powered PGx Innovations and Their Clinical Impact in Maternal and Perinatal Care.

Domain	Omics Layer	AI-PGx Innovation	Impact
Antidepressants	Genomics [28] & Metabolomics.	Neural networks optimize SSRI dosing via *CYP2D6* + *CYP2C19*.	Personalized treatment aims to reduce neonatal withdrawal.
Opioid Management	Genomics [6].	Federated learning models analyze *OPRM1* + *COMT* variants.	Influences NAS severity; promising for privacy-preserving AI.
Antiretroviral Therapy	Genomics [29] & Metabolomics [29].	Digital twins simulate fetal dolutegravir exposure.	Implies significant reduction in dosing errors.

Summary of AI-driven pharmacogenomic (PGx) applications across maternal and perinatal clinical domains, highlighting multi-omics integration and projected clinical outcomes.

**Fig. 3:**
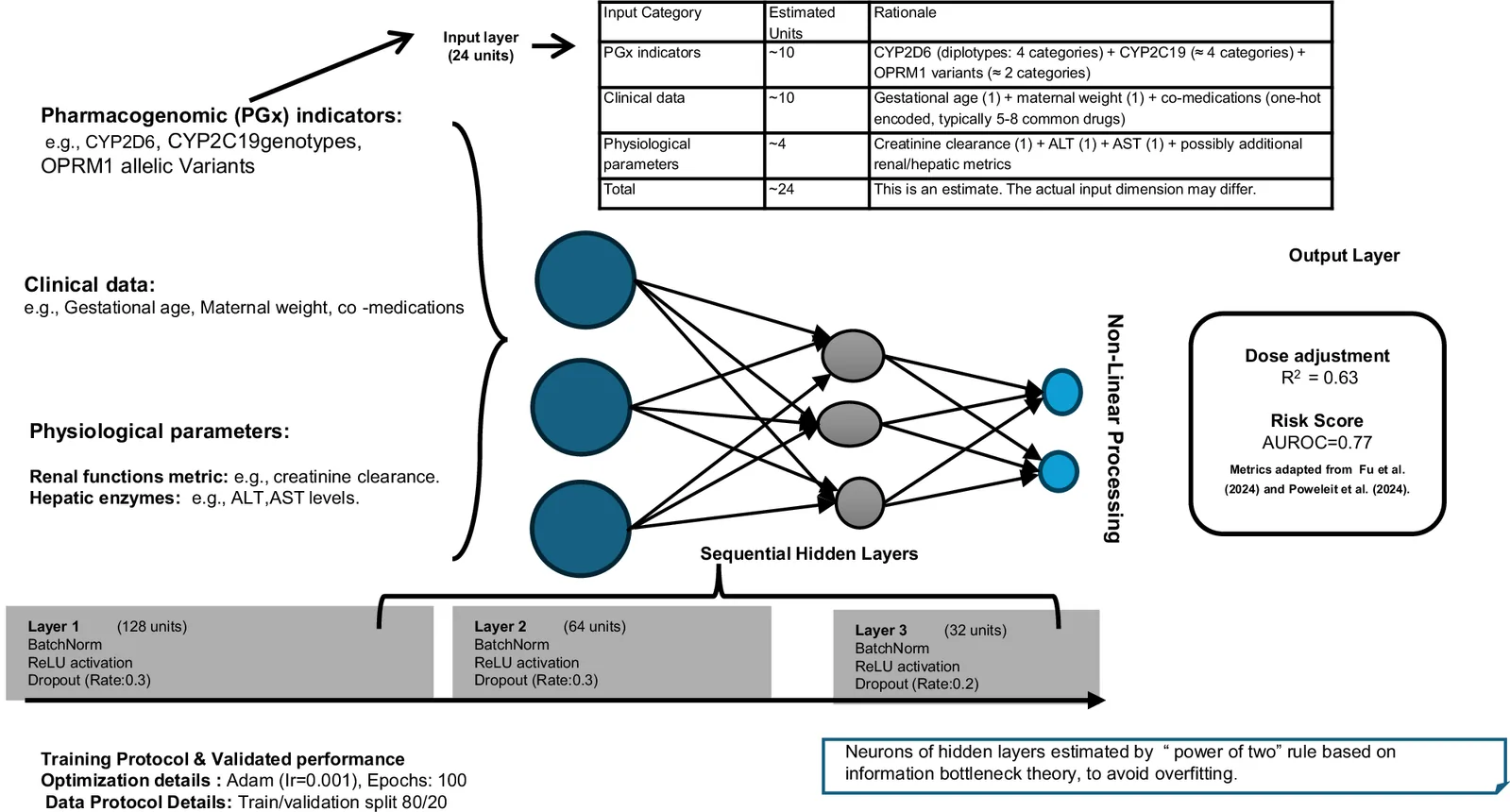
Conceptual Neural Network Architecture for Pharmacogenomic and Clinical Data Processing. Structural architecture of the DL model for dose optimization and ADR mitigation in maternal precision medicine.

Anticipated outcomes include optimization of maternal antidepressant regimens to potentially reduce adverse neonatal outcomes, such as withdrawal manifestations; early identification of high-risk patients (e.g., SSRI-poor metabolizers) and preemptive dose modification interventions. AI-PBPK frameworks, specifically those using graph neural networks, refine area-under-the-curve (AUC) drug exposure predictions with greater precision than empirical models [20, 21]. The potential value of this integrated AI approach is supported by recent studies demonstrating that AI/ML models integrating PGx and clinical data can achieve an AUROC of 0.64 for predicting sertraline side effects and up to 0.77 when predicting combined SSRI side effects [22]. Furthermore, these studies documented that AUC predictions consistently fall within two- to three-fold error ranges when validated against clinical or in vivo data, with some achieving absolute average fold errors below 2.73 for high-confidence predictions [21, 23]; however, these findings are derived from non-pregnant adult humans. To demonstrate how AI can synthesize multimodal data for personalized dosing, a conceptual deep neural network (DNN) architecture is presented that integrates pharmacogenomic indicators, clinical parameters, and physiological measurements (Fig. 3). The full architectural specifications and training protocols are detailed in Supplementary file. This architecture serves as a blueprint for translating complex biological inputs into actionable clinical guidance.

Technical constraints necessitate dataset-specific hyperparameter optimization for this conceptual architecture [24]. In the broader context of PGx, a recent review highlighted that DL models are increasingly used to map the non-linear interactions between physiological factors and genetic variants, such as those in the cytochrome P450 family, to improve the prediction of therapeutic outcomes [25]. These results demonstrate the capacity of the proposed DNN architecture to transform static genomic data into dynamic, trimester-specific therapeutic guidance. The neural network input layer is designed to process multimodal data, including maternal physiological parameters and the relevant PGx markers identified in (Tables 1 and 2). These initiatives represent key directions for future clinical impact, building on the understanding of how genetic factors influence neonatal withdrawal. Ongoing studies are quantifying the impact of AI-driven interventions on patient outcomes [14]. Simultaneously, FinnGen studies nationwide biobank data to link genotypic information with health registries for 500,000 individuals, enabling the integration of biologic and social determinants to advance personalized MNH [26, 27]. Complementing this approach on a regional scale in the Middle East and North Africa (MENA) region, the strategic framework in Qatar utilizes a centralized national electronic health record (EHR) and the Qatar Genome Program (QGP) to build representative datasets, ensuring that AI-driven precision medicine is both equitable and ethnically representative [28]. Such infrastructures are important for defining the roles of *OPRM1* and *COMT* in opioid efficacy and neonatal abstinence syndrome (NAS) risk [26, 29]. Despite these technical constraints, AI-driven approaches such as federated learning (FL) models offer a means to leverage sensitive genetic data while preserving privacy. Recent studies in adjacent healthcare domains have demonstrated the feasibility of this privacy-preserving paradigm. Validated implementations- including the atomCAT consortium’s 14-center prognostic models (c-indices 0.68–0.79) and the FedEnTrust diabetes framework (84.2% accuracy) confirm that collaborative, privacy-preserving architectures are viable for tailoring interventions in vulnerable populations [30, 31].

### Methodological advancements and privacy-preserving approaches

FL trains local AI models on decentralized datasets, aggregating parameters at a central server to build a robust global model without transmitting raw patient data [32]. In maternal-neonatal PGx, this enables the aggregation of heterogeneous genetic and clinical data to identify rare drug-gene interactions that single centers cannot adequately power [32]. However, technical challenges like data heterogeneity (non-IID) can degrade performance compared to centralized training, requiring ongoing mitigation research [31]. Ultimately, clinical implementation remains contingent on explicit regulatory frameworks and legal guidance from the FDA and EMA [33, 34].

## Pregnancy’s black box: AI’s struggle with the ultimate clinical blind spot

### From historical exclusion to algorithmic bias: a unified view

The systematic historical exclusion of pregnant and lactating populations from clinical trials has created a significant data void that directly impacts contemporary medical research. As shown in Table 3, this exclusion translates directly into algorithmic bias, compromising AI model accuracy and exacerbating health disparities when algorithms trained on non-pregnant cohorts are applied to gestation.

**Table 3:** Systemic drivers of pregnancy exclusion in clinical research and their impact on AI-driven outcomes.

Key Driver of Exclusion	Direct Consequence / Impact	Primary Data Gap / Source of Bias	AI Model Limitation / Manifestation of Bias	Clinical Health Outcome / Systemic Implication
**Ethical concerns** (fetal harm, maternal vulnerability, consent challenges)	Severe data scarcity on drug safety/efficacy in pregnancy; risk shifted to clinical context; “protected to death” inconsistency [44].	Exclusion of pregnant women from trials.	Inaccurate predictions; ineffective dosing/treatment recommendations.	Unanticipated side effects; increased maternal/fetal morbidity/mortality; delayed/inappropriate treatment.
**Historical tragedies** (*thalidomide*, *diethylstilbestrsol*)	Enactment of protective regulations limiting exposure; culture of excessive caution stalling research progress [36].	Lack of ethnic/racial diversity in cohorts.	Misdiagnosis; algorithm underestimation of optimal dosing for subgroups.	Exacerbated health disparities; less effective care for specific populations.
**Perceived legal risks / litigation fears**	Hesitancy to include pregnant women despite lack of liability claims from participation; disproportionate liability from off-label use [45].	Absence of gestational age-specific data.	Suboptimal risk assessment; inability to personalize care by stage.	Unnecessary medical interventions; missed critical warning signs.
**Logistical / operational challenges** (recruitment hurdles, lack of healthy volunteers, need for long-term follow-up, lack of regulatory support)	Therapeutic orphan- status for pregnant women [37].	Neglect of social determinants of health (SDOH).	Contextual blindness- impractical treatment recommendations.	Reduced patient adherence; erosion of trust; “trope” characterization of patients.
**Implicit Bias in AI Model Development / Evaluation**		Implicit bias in AI model development / evaluation.	The automation and perpetuation of inequitable, substandard care practices.	Widening health equity gap; creation of an “AI divide”.

### Economic and data infrastructure barriers in low-resource settings

The integration of AI into MN-PGx faces substantial economic and infrastructural barriers, particularly in low- and middle-income countries (LMICs) where the need for improved outcomes is most acute. Generating comprehensive multi-omics data demands significant financial investment, as each distinct layer: genomics, transcriptomics, proteomics, metabolomics, epigenomics, requires specialized platforms, sophisticated laboratory techniques, and high-throughput sequencing equipment [25, 35]. Although next-generation sequencing has made individual data points more accessible, the sheer scale of multi-omics research remains inherently expensive, demanding substantial start-up capital for equipment and consistent operational budgets for reagents and skilled personnel [25]. This cost structure presents a major barrier to entry for many healthcare systems, exacerbating existing disparities in access to advanced medical technologies [36]. Maternal mortality rates associated with chronic conditions are nearly three times higher in such settings than in high-income countries, a disparity primarily attributable to inadequate prenatal care and delayed medical interventions [37]. India’s Garbhini-GA2 model, which uses standard ultrasound biometry and machine learning, reduced gestational age error threefold compared to Western standards, demonstrating that representative data, not AI complexity, is the key LMIC barrier [38]. The challenge therefore extends beyond the direct costs of AI implementation to encompass the fundamental absence of essential healthcare resources and robust health systems- prerequisites without which effective AI integration remains unattainable. This dilemma is especially acute in regions such as the MENA region, where AI applications that rely on complex DL are fundamentally “data hungry” [39]. Without massive, targeted investment in foundational genomic infrastructure and data collection initiatives, AI-driven PGx will remain a theoretical framework unable to progress beyond models trained on non-representative Western cohorts [40]. The necessity for region-specific data is underscored by significant pharmacogenomic diversity: CYP2D6 ultrarapid metabolizer prevalence is 20–29% in East-African populations (including Ethiopia) and 16–40% in North African groups (e.g., 39.5% in Mozabite Algerians), compared to only 2–3% in Europeans and 0–3% in East Asians. This variation presents a critical risk of dose-related toxicity or therapeutic failure if non-representative Western models are applied [41]. AI solutions require deployment in regions where basic maternal healthcare infrastructure-including reliable electricity, internet connectivity, and trained personnel is often limited, underscoring the need for implementation strategies that account for these practical constraints [42]. Addressing these foundational economic and infrastructural deficits is therefore essential for ensuring that AI-driven PGx can genuinely contribute to equitable maternal-neonatal health outcomes globally.

## Future directions & solutions and limitations

Addressing the identified challenges and fully realizing the potential of AI in MN-PGx requires concerted efforts across several key domains, including advancements in data management, technical methodologies, and policy frameworks.

### Data standardization and harmonization for multi-omics integration

Effective AI integration requires standardized multi-omics datasets to ensure consistency and compatibility across disparate platforms. Currently, multi-omics data often originate from different labs and platforms, leading to variations in format, quality, and annotation [43]. While AI is adept at processing complex datasets, these underlying inconsistencies can limit the accuracy and generalizability of the AI models. Therefore, a key future direction involves establishing robust data standardization and harmonization protocols. This means developing common standards for data collection, formatting, and description, alongside effective quality control measures. Collaborative efforts to create standardized and accessible data repositories are important to ensure interoperability and comparability across different omics layers and datasets [44]. Addressing this fundamental challenge is important to realize the potential of AI-driven multi-omics for precision medicine in MN-PGx and beyond. While achieving data interoperability is a foundational requirement, the utility of standardized multi-omics datasets is ultimately defined by the integrity of the models they inform; consequently, establishing harmonized data architectures provides the necessary substrate to address the historical exclusion of pregnant populations through the algorithmic bias mitigation strategies discussed in the following section.

### Strategies for algorithmic bias mitigation

Mitigating algorithmic bias in MNH-AI systems begins with addressing the data void created by the historical exclusion of pregnant individuals from clinical trials as briefly discussed in (Table 3). Generative Adversarial Networks (GANs) provide a robust framework for augmenting datasets and bypassing the ‘pregnancy black box’ [45, 46]. Furthermore, the application of transfer learning enables the translation of established pharmacological insights from general adult populations into the maternal-neonatal domain. This approach allows models to leverage broad systemic knowledge while fine-tuning parameters to account for the unique pharmacokinetic and pharmacodynamic shifts inherent in pregnancy, thereby optimizing accuracy despite limited domain-specific data. These data-centric efforts are most effective when combined with transfer learning and meta-learning, which allow models to generalize from larger, non-pregnant populations to smaller, pregnancy-specific cohorts [47]. Complementing these approaches, explainable AI methods empower clinicians to understand how predictions are made and to identify discriminatory patterns that might otherwise remain hidden, thereby building clinical trust while facilitating the detection of bias [48]. However, none of these technical strategies can replace the foundational need to include pregnant and lactating individuals in clinical research-the only way to generate unbiased, representative data that serves all populations, irrespective of race, ethnicity, socioeconomic status, or geography [39, 49].

### Navigating the ethical minefield: policy solutions

While technical advancements in data synthesis and algorithmic refinement can improve model accuracy, they do not inherently resolve the broader societal risks associated with large-scale genomic data integration. The integration of AI-driven PGx into MNC presents significant ethical and regulatory challenges that must be addressed through coherent policy frameworks. A central concern is informed consent for fetal pharmacogenomic testing. Informed consent requires transparency regarding direct risks, reproductive implications, and data-sharing protocols [50, 51]. This requires transparent communication about how data will be collected, shared, and used, along with clear assurances that sensitive information will not be misused or disclosed without explicit consent [52]. Beyond clinical utility, the aggregation of maternal-fetal data introduces significant geopolitical risks. Genetic surveillance could be repurposed for immigration enforcement, predatory insurance premium adjustments, or population-level tracking of pregnancy outcomes [53–57]. Such ‘function creep’ creates a systemic disincentive and a chilling effect that deters care-seeking among historically disenfranchised communities [46, 55, 58]. To mitigate these risks, governance should center on three pillars: legislative firewalls prohibiting non-medical data repurposing, revocable consent mechanisms that ensure patient autonomy, and adherence to OECD 2023 health data standards [59–62]. Implementing these protections, alongside restoring the limited public charge rule, is important for addressing the barriers to equitable precision medicine [33, 55].

### Policy & collaboration

The successful translation of AI-driven PGx from theoretical models to bedside MNC is contingent upon a robust and harmonized policy landscape. Central to this evolution is the establishment of global pregnancy PGx registries, which serve as the data backbone for large-scale research. Building on initiatives like NIH PRGLAC, international collaborations facilitate the systematic sharing of de-identified clinical data [63]. As highlighted in recent frameworks, utilizing algorithms like ‘FedAvg’ enables individual hospitals to refine global models on their proprietary genomic data while maintaining strict compliance with privacy regulations such as GDPR and HIPAA [32]. This approach is essential for capturing the broad ancestral diversity required for global precision medicine, ensuring that AI models are not merely optimized for high-income cohorts but are representative of the global maternal population [64]. Beyond data collection, the policy environment must actively incentivize pharmaceutical industry inclusion. Historically, the exclusion of pregnant and lactating individuals from clinical trials has been driven by perceived legal risks and ethical caution. To bridge the data gaps identified earlier, stakeholders should transition from a culture of protective exclusion to one of evidence-based inclusion, utilizing emerging regulatory pathways-such as the FDA’s recent frameworks for decentralized trials and real-world evidence (RWE)-to incentivize the enrollment of pregnant populations [34]. However, emerging regulatory pathways and ethical frameworks now provide a structured mechanism for companies to voluntarily include these populations, thereby generating the foundational datasets necessary for AI-driven personalized medicine [34, 65]. This shift is complemented by the need for effective integration with EHR and obstetric workflows. For AI-PGx tools to be clinically viable, they must be embedded within existing digital infrastructures in a manner that is user-friendly and capable of delivering real-time decision support during routine patient care [28, 66]. Recent trends emphasize that the scalability of these interventions depends less on the complexity of the algorithms and more on the readiness of health systems to incorporate them into daily practice [67]. Finally, achieving global health equity requires dedicated funding mechanisms and governance structures. To address the stark resource disparities between high-income and low-resource settings, a “Global AI-PGx for MNH Fund” could pool resources from multilateral agencies like the WHO and the World Bank to build essential genomic infrastructure in underserved regions [68]. Public-private partnerships (PPPs) and innovative financing models, such as data impact bonds, also play a vital role in incentivizing the generation of representative multi-omics data [69]. These mechanisms are crucial for transitioning the economic burden away from LMICs, ensuring they are active participants in the global data economy rather than mere subjects of data extraction. By fostering international trust and cooperation among health regulators, as outlined in the WHO-roadmap, the global community can ensure that the benefits of AI-driven precision medicine are distributed equitably across all populations [70].

### Enhancing equitable access to precision medicine in low-resource environments

The ultimate goal of integrating AI into maternal-neonatal PGx (MN-PGx) is to ensure equitable access to precision medicine, particularly in low-resource settings where the need is acute. AI-enabled telemedicine and virtual assistant technologies are important for bridging healthcare disparities affecting underserved and rural populations [71]. AI applications facilitate remote pregnancy monitoring, genetic screenings, and continuous monitoring of maternal health parameters with real-time alerts for deviations [72]. This capability directly addresses the “last mile” problem in healthcare delivery by providing decentralized precision medicine directly to underserved populations. This has the potential to transform global health equity by overcoming geographical barriers leading to improved outcomes for historically disenfranchised populations.

### Personalized medicine charting and decision support systems (CDS)

Translating complicated data into actionable clinical guidance for decision-making has potential value; however, it remains a significant challenge. Building upon the need for precision medicine in MNC, a conceptual framework for personalized patient charts emerges as a key tool for translating complex PGx and clinical data into intuitive, actionable therapeutic guidance. The framework integrates various data streams such as genetic information, clinical parameters, physiological variables, and multi-omics data to provide individual recommendations [25]. The successful clinical translation of AI-driven PGx remains contingent on overcoming significant integration barriers within EHR environments. A recent scoping review (2024) highlights that while PGx is currently the most mature implementation domain for precision medicine, technical gaps specifically the ‘paucity’ of integrated social determinants of health (SDoH) and the lack of computable standardized formats for multi-omics data hinder real-time clinical application [73]. The integration of multi-omics data into such frameworks is supported by the work of Mumcu et al. [44], demonstrated that combining placental metabolomics and transcriptomics data in preeclampsia reveals significant disruptions in metabolic pathways (e.g., the Krebs cycle, amino acid metabolism) and identified differentially expressed genes linked to angiogenesis and inflammation. The work demonstrates how multi-omics datasets can be generated and analyzed to provide a mechanistic understanding of disease, generating the type of high-dimensional molecular data that can serve as input for AI-driven CDS tools [44]. Notably, the study found that a gene interaction network derived from multi-omics data yielded significantly stronger protein-protein interaction enrichment compared to a network based on transcriptomics alone, underscoring the importance of integrating multiple omics layers to achieve a comprehensive view of disease pathophysiology, a prerequisite for accurate risk prediction and tailored therapeutic intervention. Adding this molecular perspective, the integration of SDoH into CDS frameworks is advanced by Soley et al. [74], who demonstrated the feasibility of using natural language processing (NLP) to extract key SDoH factors such as social support, occupation, and substance use from unstructured EHR notes. Their study showed that models like ClinicalBERT could achieve high performance in identifying these factors. Crucially, their regression analysis provided quantitative evidence of the clinical relevance of SDoH documented substance use was associated with significantly increased odds of pregnancy complications, while the presence of social support was strongly protective. These findings demonstrate that SDoH factors carry predictive signals comparable to traditional clinical biomarkers and can be systematically rendered computable through AI, enabling their integration into personalized CDS frameworks [74]. Optimization of CDS systems requires addressing the ‘literacy barrier’ among providers through embedded educational materials and ‘active alerts’ to ensure recommendations are actionable at the point of care [75]. These personalized charting systems are further enhanced by remote monitoring capabilities, which provide real-time data inputs that enable dynamic updates to dosing recommendations and risk assessments throughout gestation. The clinical utility of this remote monitoring framework is supported by recent cross-country analyses and systematic reviews; the findings indicate that AI-empowered mobile health (mHealth) interventions can correct up to 27% of deviations from long-term maternal mortality trends annually by enabling early detection of high-risk conditions such as preeclampsia and gestational diabetes [76]. Furthermore, wearable health-tracking technologies integrated with AI-driven analytics have demonstrated the ability to monitor maternal cardiovascular parameters and physical activity with high fidelity, providing real-time alerts that facilitate timely clinical interventions in underserved regions [77]. These technologies not only bridge geographical barriers but also enhance health-seeking behaviors by providing personalized, non-invasive feedback to expectant mothers [78].

### Realistic data requirements and phased implementation

The ambition of AI-driven PGx must be grounded in the practical realities of data volume, quality, and curation. Developing clinically robust models for maternal health is not merely a matter of algorithmic sophistication; it requires massive, high-quality datasets that can capture rare drug-gene interactions and the intricate physiological shifts unique to pregnancy [3]. Current empirical scaling laws for multi-omics integration suggest that to adequately power the discovery and validation phases accounting for the non-linear interactions between polygenic risk and dynamic gestational changes researchers typically require cohorts exceeding 20,000–30,000 deeply phenotyped individuals [40]. These data requirements naturally vary depending on the complexity of the clinical task at hand. For instance, optimizing dosing for well-understood single-gene and drug pairs, such as the relationship between *CYP2D6* variants and SSRIs, might be validated with prospective datasets of approximately 500 individuals per metabolizer phenotype [79]. However, moving toward polygenic risk stratification for complex conditions like neonatal abstinence syndrome or preeclampsia demands a significantly larger scale, typically requiring 2000–5000 participants with longitudinal follow-up to ensure the models are generalizable across populations [80]. The most advanced goal-integrating genomic, transcriptomic, and proteomic data for trimester-specific dose prediction presents a high- dimensional challenge where sample sizes below 15,000 carry a severe risk of overfitting, which can lead to poor clinical performance [81, 82]. Given these steep requirements, a phased implementation strategy is not just a rational choice but clinically advisable. This approach recognizes the fundamental differences in how various ML models handle data. Simpler, more interpretable models like Random Forest or XGBoost can often deliver immediate clinical value with smaller cohorts of 500–1000 patients, making them ideal for initial single-gene validation and building foundational trust among clinicians [79], as evidenced by recent implementations of gradient-boosting systems for pharmacogenetic recommendations [83]. In contrast, clinical-grade DL models for complex, multi-omics integration require datasets exceeding 10,000–15,000 deeply phenotyped individuals to mitigate algorithmic bias and ensure generalizability [81]. Consequently, the initial phase of implementation should focus on these more accessible models to establish evidence-based PGx protocols. As these foundations are laid, investment must then pivot toward assembling large-scale international collaborations, such as a Global Pregnancy Collaboration (CoLab), to reach the necessary thresholds for more complex deep-learning applications [40]. Without a massive, coordinated investment in foundational genomic and clinical data infrastructure, particularly in underrepresented global populations, complex AI applications are likely to remain theoretical for most of the world [40]. The resource-limited settings require an implementation approach that needs to be executed in several stages. The approach creates tangible benefits from the start while building the essential data infrastructure needed for more advanced applications.

### Critical limitations and future research priorities

Primary limitations beyond the ‘data void’ include a lack of randomized trials, the absence of standardized performance metrics, and the technical complexity of integrating multi-omics into existing clinical workflows [52]. Research should shift its focus from developing theoretical models to studying the practical application of these models through prospective testing in clinical environments. Addressing these limitations requires a dedicated translational research agenda that will lead to clinical implementation and equitable healthcare access. The research agenda prioritizes two complementary directions. First, comparative effectiveness studies are needed to evaluate AI-PGx performance against standard medical practices [9]. Second, international FL consortia should include underrepresented populations from MENA and African cohorts to achieve global applicability [32, 40].

## Conclusion

The transition from the ‘pregnancy black box’ to AI-supported precision care requires a multi-stakeholder framework focused on inclusive clinical trials and global federated data networks. AI’s integration of multi-omics and predictive modeling holds significant potential to reduce adverse reactions and optimize maternal dosing. Among the emerging issues in the field are deep-learning models for preeclampsia prediction, digital twin generation for simulating fetal drug exposures, and privacy-preserving FL paradigms. Furthermore, the economic and resource-intensive nature of multi-omics data generation and AI infrastructure poses substantial barriers, particularly in low-resource settings where the need for improved maternal-neonatal outcomes is most acute.

**Fig. 4:**
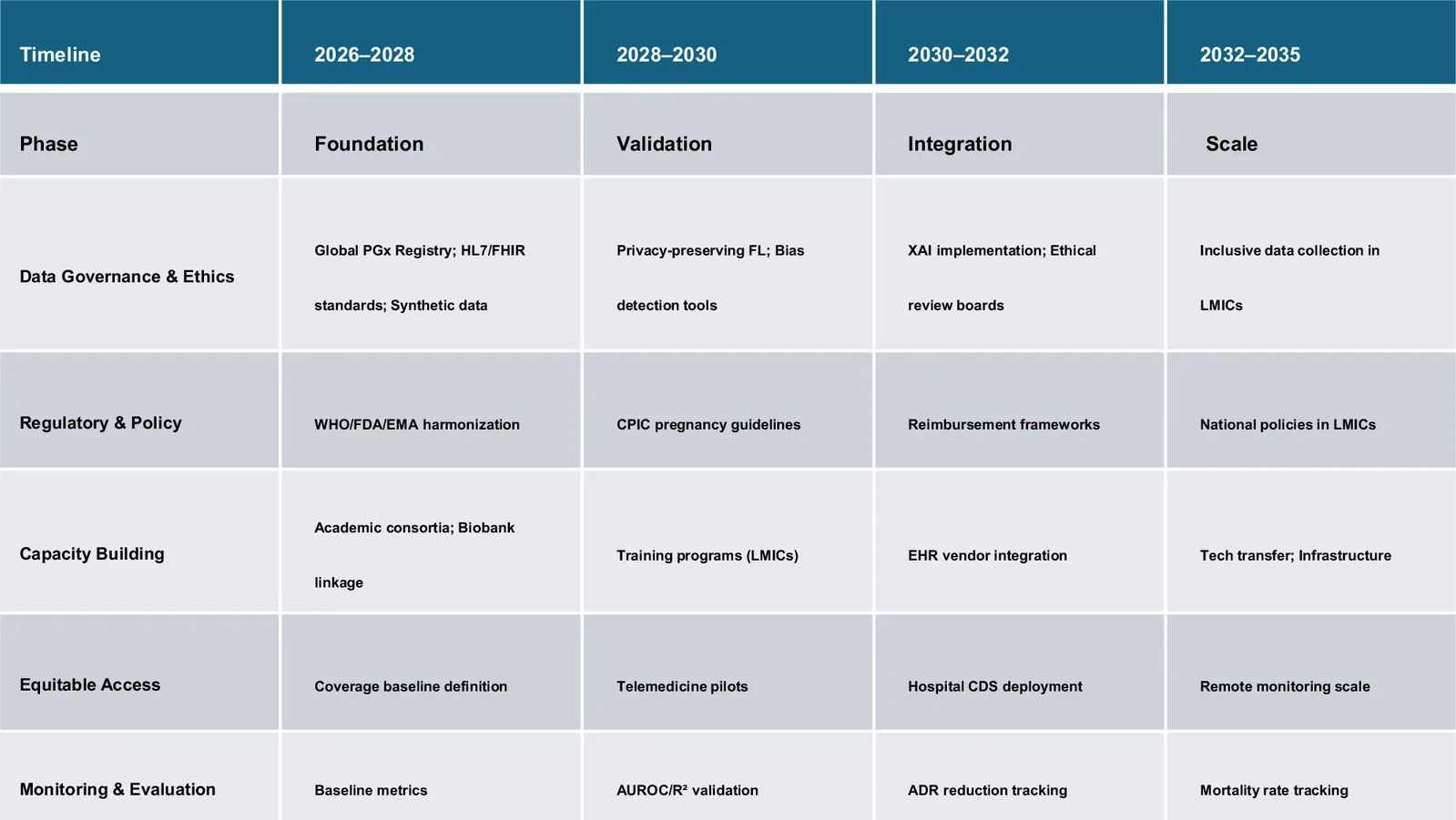
Strategic Roadmap for AI-Powered Pharmacogenomics. The roadmap spans four phases (Foundation, Validation, Integration, Scale) across five key pillars: data governance and ethics, regulatory and policy harmonization, capacity building, equitable access, and monitoring/evaluation.

The proposed roadmap (Fig. 4) requires proactive strategies for algorithmic bias mitigation, including innovative data augmentation techniques and the development of fairness-aware AI algorithms. The roadmap is not purely theoretical. Table 1 illustrates emerging technical feasibility through global registries and high‑performance predictive models, though most require further external validation and Table 4 provides empirical evidence supporting each roadmap phase, with corrected citations for interoperability and predictive performance. Proceeding without robust policy and regulations to navigate this ethical minefield could perpetuate risks associated with fetal PGx testing and data privacy. The concrete timelines follow a 10-year strategic framework. The transition from retrospective exploration to robust clinical implementation is evidenced by the successful integration of AI-powered PGx insights into EHRs, which have been shown to reduce medication errors and optimize therapeutic outcomes in MNC supporting the roadmap figure [84]. Additionally, (Table 4) demonstrates that each stage of the roadmap from data standardization to global equitable access is supported by measurable gains in diagnostic accuracy, predictive performance, and health system efficiency.

**Table 4:** Empirical evidence and performance metrics validating the strategic roadmap for AI-powered maternal pharmacogenomics.

Roadmap Phase	Empirical Evidence / Key Metric	Relevance to Roadmap
Data Governance & Ethics”	Integration of PGx data (e.g., CYP2C19, CYP2D6) into Epic’s Genomic Module using HL7 version 2 (HL7v2) standards successfully reduces manual entry errors and supports complex genomic elements, including star alleles, phenotypes, activity scores, and variant-level discrete data [95]. Furthermore, the German genomDE study confirms that FHIR genomics reporting and genomics alliance for global health (GA4GH) Phenopacket schemas enable broad genomic data representation and provide a framework for international interoperability [96].	These metrics validate that established HL7 messaging standards (HL7v2) enable reliable, granular PGx data integration into EHRs-a critical building block that supports the broader HL7/FHIR framework outlined in the roadmap for global interoperability.
Regulatory &Policy	With discordance rates of 68–71% between FDA and EMA pregnancy labeling highlighting an urgent need for regulatory harmonization, policy momentum is growing [42]. This is evidenced by the July 2025 FDA E21 guidance mandating the inclusion of pregnant and breastfeeding individuals in clinical trials, alongside WHO consultations aimed at addressing historical infrastructure gaps [43].	This evidence underscores the necessity of regulatory alignment and demonstrates the emerging policy momentum required to dismantle the “pregnancy black box” and support AI-PGx implementation globally.
Capacity building	The FedEnTrust framework has validated the feasibility of privacy-preserving FL, achieving 84.2% accuracy for diabetes prediction across diverse populations [40]. Additionally, Qatar’s national strategy integrates a centralized EHR with the Qatar Genome Program, building representative datasets and demonstrating institutional capacity for EHR‑vendor integration and AI‑driven precision medicine [38].	These milestones confirm that privacy‑preserving FL architectures are scalable for cross‑institutional collaboration, while national‑level EHR–genomics integration (Qatar) validates the roadmap’s capacity‑building goals for EHR vendor integration and infrastructure readiness.
Equitable access	In LMICs, the implementation of AI-driven mHealth interventions are associated with a 27% reduction in mortality after correcting for cluster-level trends [86].	This quantitative evidence validates that AI-driven interventions can significantly improve maternal health outcomes in resource-limited settings, thereby supporting the roadmap’s equity goals.
Monitoring & Evaluation	Predictive models integrating clinical and therapeutic drug monitoring data have achieved an R² of 0.63 for drug concentration forecasting [97]. Additionally, DrugGPT has demonstrated 17.3–39.8% higher accuracy in adverse event prediction compared to existing models [9].	These metrics validate the predictive performance of AI‑PGx models, supporting the need for continuous monitoring and evaluation as outlined in the roadmap.

The ultimate goal is reducing global maternal mortality and achieving the SDG targets. Such advancements are important for fulfilling the mandate of Sustainable Development Goal 3 (SDG 3), specifically by providing the technical framework necessary to achieve Target 3.1 (reducing global maternal mortality) and Target 3.2 (ending preventable deaths of newborns and children under 5 years of age) by 2030. While the proposed roadmap provides a framework for global collaboration, regional initiatives demonstrate how national policies can operationalize key pillars. For instance, an analysis of Qatar’s strategic AI trajectory in healthcare illustrates how frameworks like Qatar National Vision 2030 can advance data governance and infrastructure to move PGx from research toward clinical implementation [28]. Some key points for global collaboration would include international PGx registries and incentives for pharmaceutical companies to include pregnant subjects in clinical trials, as this will form the basis for basic and diverse datasets for unbiased AI training. Finally, with the help of AI-driven remote monitoring and telemedicine solutions, a novel way to provide precision medicine even in low-resource settings can occur, thereby bridging healthcare gaps and improving health outcomes worldwide. Dismantling the ‘pregnancy black box’ through AI-driven processes is a realistically achievable goal within the decade, provided the research community adopts the coordinated roadmap proposed herein. A multi-stakeholder framework targeting three priorities is needed for the transition. An important first step is improving clinical trial design to require the inclusion of pregnant and lactating people. Aligning with emerging FDA/EMA regulatory guidance seeks to overcome the historic exclusion policies to remedy data shortages [34, 65]. At the same time, there is an urgent need for global federated data networks for privacy-preserving computational modeling in the federated global ancestral cohorts. Facilitating the establishment of these data networks will necessitate a global collaboration involving researchers and industry players as well as maternal-child groups [32]. Finally, there is a need for developing and validating fairness-aware AI models and XAI tools specifically for the maternal-neonatal dyad [85]. By bridging these gaps, the research community can achieve a future of precision pharmacotherapy that provides safe, efficacious, and tailored therapeutic interventions for mothers and neonates.

## Supplementary information


Supplementary file

