# Supplementary material for: Beyond the ‘Pregnancy Black Box’: a global roadmap for artificial intelligence-driven pharmacogenomics in maternal-neonatal health
Source: Pharmacogenomics J. 2026 Jun 22;26(4):29. doi: 10.1038/s41397-026-00422-4 (PMC13286999; doi:10.1038/s41397-026-00422-4)
Supplement: Supplementary file 1 — Supplementary file [file 41397_2026_422_MOESM1_ESM.docx]

**2. Methodology: Literature Search Strategy**

This is a narrative conceptual review; therefore, formal risk‑of‑bias assessment of individual studies was not performed.

**2.1. Data Sources and Search Strategy**

A comprehensive search using multiple strategies was performed on various databases and platforms (e.g., PubMed, Scopus, Web of Science and Google Scholar). The search strategy keywords centered on three core concepts: (1) Maternal-Neonatal Health (2) Pharmacogenomics and (3) Artificial Intelligence. The search terms were combined using Boolean operators (AND, OR) as follows:

Domain 1: ("Pregnancy" OR "Maternal Health" OR "Perinatal" OR "Neonatal" OR "Obstetrics").

Domain 2: ("Pharmacogenomics" OR "PGx" OR "Pharmacogenetics" OR "Drug Response").

Domain 3: ("Artificial Intelligence" OR "AI" OR "Machine Learning" OR "Deep Learning" OR "Federated Learning").

The final search string used was: ("Pregnancy" OR "Maternal Health" OR "Perinatal" OR "Neonatal" OR "Obstetrics”) AND ("Pharmacogenomics" OR "PGx" OR "Pharmacogenetics" OR "Drug Response") AND (“Artificial Intelligence" OR "AI" OR "Machine Learning" OR "Deep Learning" OR "Federated Learning”.

**2.2. Study Selection and Eligibility Criteria**

The initial search results were screened for relevance based on the title and abstract. Full text articles were then retrieved and assessed against the following eligibility criteria:

| **Criterion** | **Inclusion** | **Exclusion** |
| --- | --- | --- |
| Publication Type | Original research articles, systematic reviews, meta-analysis, authoritative review articles and policy/guidance documents (e.g., FDA). | Conference abstracts, and non-peer-reviewed articles (preprint). |
| Language | English language publications only. | Non-English language publications. |
| Relevance | Must explicitly discuss the application of AI/Machine Learning to PGx or drug safety/efficacy within the context of pregnancy, maternal, or neonatal health. | Studies focused solely on general PGx or general AI in healthcare without a specific maternal-neonatal focus. |

**2.3. Data Extraction and Synthesis:**

The main aim of this conceptual review is to emphasize the unpredictable pharmacokinetic profiles during pregnancy and the promise of AI in real-world applications for precision medicine in perinatal care. The review extracts data on AI models used with PGx or omics data to predict maternal conditions like preeclampsia and determine personalized dosing. The included studies measured model performance using established metrics such as accuracy and AUC.

- **Technical Proposal for Conceptual Deep Neural Network Architecture**: The architecture is representative; specific layer dimensions and hyperparameters require optimization for each application and dataset. The network accepts three parallel data streams at the input layer. First, clinical data encompasses trimesters of pregnancy, maternal age, and weight, capturing the dynamic physiological changes across gestation that profoundly impact drug distribution and clearance. Physiological measurements include renal function (eGFR) and hepatic enzyme activity, which undergo marked pregnancy-associated alterations. For example, renal clearance increases by approximately 35–50% by the third trimester, and CYP3A4 activity is enhanced by 45–65% during pregnancy (1, 2). Pharmacogenomic markers include CYP2D6 metabolizer status, CYP2C19 metabolizer status, and OPRM1 variants, which are key determinants of drug metabolism and opioid response (3-5). Second, three sequential hidden layers progressively extract and integrate features from these input streams. The first two layers each employ ReLU (Rectified Linear Unit) activation followed by dropout regularization at a rate of 0.3 and batch normalization. ReLU introduces essential non-linearity while mitigating vanishing gradient problems common in deep networks. Dropout randomly deactivates 30% of neurons during training to prevent co-adaptation and overfitting, a technique originally validated by Srivastava and colleagues (6), while batch normalization stabilizes learning by normalizing layer inputs to accelerate convergence and reduce sensitivity to initialization (7). The third hidden layer similarly applies ReLU activation but with a slightly reduced dropout rate of 0.2, reflecting diminished regularization requirements as features become more concentrated and task-specific closer to the output layer. The number of neurons in each hidden layer is deliberately unspecified, as optimal dimensions are dataset-dependent and determined through hyperparameter optimization tailored to each application's specific feature dimensionality and sample size (8). The output layer generates two complementary clinical predictions through multi-task learning, which enhances generalization by leveraging shared representations across related tasks. A continuous dose recommendation in milligrams per day is produced through linear activation, providing trimester-specific optimal dosing. Finally, a probabilistic risk score between zero and one is generated through sigmoid activation, quantifying the predicted risk of adverse outcomes including neonatal withdrawal syndrome or maternal side effects, which can serve as a threshold for clinical alerting. The network is trained using an optimization protocol validated across pharmacogenomic and biomedical deep learning applications. The loss function combines mean squared error for dose prediction with binary cross-entropy for risk classification, enabling simultaneous optimization of both regression and classification objectives (7). The Adam optimizer with a learning rate of 0.001 adaptively adjusts parameter updates and has become standard in clinical prediction modeling; this method was originally described in a 2015 conference paper and has been extensively validated in peer-reviewed studies (7, 9). Training proceeds with a batch size of 32 for up to 100 epochs, incorporating early stopping with patience of 10 epochs to halt training when validation performance ceases to improve, thereby preventing overfitting while ensuring adequate learning (7, 10).

**References**

1. Dallmann A, Pfister M, van den Anker J, Eissing T. Physiologically Based Pharmacokinetic Modeling in Pregnancy: A Systematic Review of Published Models. Clinical Pharmacology & Therapeutics. 2018;104(6):1110–24.10.1002/cpt.1084

2. Ismail MA. Integrating pharmaco-multiomics and AI for precision perinatal psychiatry: A call for dynamic dosing. Asian Journal of Psychiatry. 2026;117:104834.10.1016/j.ajp.2026.104834

3. Yen E, Gaddis N, Jantzie L, Davis JM. A review of the genomics of neonatal abstinence syndrome. Frontiers in Genetics. 2023;14:1140400.10.3389/fgene.2023.1140400

4. Athreya AP, Neavin D, Carrillo-Roa T, Skime M, Biernacka J, Frye MA, et al. Pharmacogenomics‐Driven Prediction of Antidepressant Treatment Outcomes: A Machine‐Learning Approach With Multi‐trial Replication. Clinical Pharmacology and Therapeutics. 2019;106(4):855.10.1002/cpt.1482

5. Poweleit EA, Vaughn SE, Desta Z, Dexheimer JW, Strawn JR, Ramsey LB. Machine learning-based prediction of escitalopram and sertraline side effects with pharmacokinetic data in children and adolescents. Clinical pharmacology and therapeutics. 2024;115(4):860.10.1002/cpt.3184

6. Srivastava N, Hinton G, Krizhevsky A, Sutskever I, Salakhutdinov R. Dropout: a simple way to prevent neural networks from overfitting. Journal of Machine Learning Research. 2014;15(1):1929–58.10.5555/2627435.2670313

7. Almufadi NF, Alhasson HF, Alharbi SS. E-DFu-Net: An efficient deep convolutional neural network models for Diabetic Foot Ulcer classification. Biomolecules & Biomedicine. 2025;25(2):445–60.10.17305/bb.2024.11117

8. Narykov O, Zhu Y, Brettin T, Evrard YA, Partin A, Xia F, et al. Data imbalance in drug response prediction: multi-objective optimization approach in deep learning setting. Briefings in Bioinformatics. 2025;26(2):bbaf134.10.1093/bib/bbaf134

9. Reyad M, Sarhan AM, Arafa M. A modified Adam algorithm for deep neural network optimization. Neural Computing and Applications. 2023;35(23):17095–112.10.1007/s00521-023-08568-z

10. Blaser MJ. A Framework for Effective Application of Machine Learning to Microbiome-Based Classification Problems. mBio. 2020
